# Visual orientation discrimination in adults with ADHD and ASD: the differential impact of clinical diagnosis and trait severity

**DOI:** 10.3389/fpsyt.2026.1754032

**Published:** 2026-02-23

**Authors:** Vesko Varbanov, Paul G. Overton

**Affiliations:** Department of Psychology, School of Psychology, University of Sheffield, Sheffield, United Kingdom

**Keywords:** ADHD, ASD, clinical diagnosis, neurodevelopmental disorders, sensory processing

## Abstract

**Objectives:**

This study aimed to clarify whether clinical diagnosis, as opposed to trait severity alone, differentiates sensory processing profiles in adults with ADHD and ASD. Specifically, we compared clinical and non-clinical cohorts matched on standardized self-report measures to test the impact of diagnosis on visual orientation discrimination.

**Methods:**

A total of 152 adults were assigned to four matched groups: clinical ADHD, non-clinical ADHD, clinical ASD, and non-clinical ASD (n = 38/group). ADHD and ASD traits were assessed using the Adult ADHD Self-Report Scale (ASRS) and Broad Autism Phenotype Questionnaire (BAPQ). Sensory performance was measured using a visual orientation discrimination task (with vertical and oblique conditions). Propensity score matching and ANCOVAs (controlling for age/gender) were used to ensure comparability between the clinical and non-clinical groups.

**Results:**

Clinically diagnosed ADHD participants displayed significantly poorer oblique orientation sensitivity than non-clinical controls, while clinical ASD participants exhibited superior vertical orientation discrimination compared to their matched non-clinical group. No significant differences were found for vertical thresholds in ADHD and oblique thresholds in ASD. These patterns remained after controlling for age and gender, indicating disorder-specific sensory trajectories, with bidirectional effects of severity and/or clinical diagnosis on sensory performance.

**Conclusions:**

Findings suggest that clinical diagnosis captures qualitative as well as quantitative differences not reflected by trait severity alone. Within visual orientation discrimination, ADHD and ASD showed different patterns of diagnostic modulation that are consistent with dimensional and dimensional-categorical (hybrid) interpretative frameworks, respectively.

## Introduction

According to the *Diagnostic and Statistical Manual of Mental Disorders* (5th ed.; DSM-5; 1), Attention Deficit/Hyperactivity Disorder (ADHD) is defined by impairments in attentional control and by excessive motor activity expressed through hyperactivity and impulsivity (2, 3). This contrasts with Autism Spectrum Disorder (ASD), which the DSM-5 describes as involving deficits across three key domains: language ability, repetitive and inflexible behaviors, and social interaction (1). Although these presentations appear distinct, and indeed were mutually exclusive diagnoses prior to the DSM’s 5th edition (4, 5), both disorders share overlapping genetic underpinnings (6). Consequently, recent research has increasingly examined their co-occurrence, suggesting that the two not only interact (7) but may also intensify one another’s symptoms (8). This question of shared mechanisms has become increasingly salient as growing numbers of individuals experiencing challenges in academic, occupational, and social functioning are being diagnosed with one or both conditions (9), particularly within adult populations, where research remains limited (10, 11).

Atypical sensory responses are highly prevalent in both ADHD and ASD and are linked to shared patterns of neural connectivity (12, 13), including alterations in primary sensory regions and attentional networks, with overlapping atypical sensory symptoms and neural mechanisms implicated in bottom-up and top-down brain processes central to both disorders (14, 15). This highlights sensory processing as a transdiagnostic marker and thus a sensible construct that can help understand whether – in spite of the categorical clinical picture of the disorders - ADHD and ASD emerge from a shared common neural substrate (16–20). Evidence across both conditions indicates comparable general, high level difficulties in the detection, modulation, and subsequent internal organization of sensory information (21). Such impairments can give rise to atypical (either heightened or reduced) sensitivity across multiple sensory modalities (22, 23), often resulting in challenges adapting to environmental demands and engaging in everyday tasks (24, 25). However, the question still remains of whether ADHD and ASD give rise to comparable sensory changes at a more granular level of analysis.

Recent research by Varbanov et al. (26) investigated the capacity to accurately distinguish between different orientations of sinusoidal gratings, called Visual Orientation Discrimination (VOD), in non-clinical cohorts with high levels of ADHD and ASD-like traits and found similar levels of reduced sensory sensitivity in both (in line with 16, 17, 27). However, in contrast to Varbanov et al. (26), other studies investigating VOD have reported that individuals with a clinical diagnosis of ASD demonstrate enhanced ability to discriminate oblique orientations compared to neurotypical individuals (28). Likewise, Dickinson et al. (29) reported higher levels of ASD traits were associated with better VOD performance in a non-clinical ASD cohort, whilst others have found no significant relationship between non-clinical ASD traits and visual discrimination thresholds (30).

The seemingly contradictory research on VOD just discussed adds unwanted confusion to the issue of a possible sensory commonality between ADHD and ASD and its contribution to our understanding concerning the crucial question of whether ADHD and ASD are a product of a common neural substrate (as posited by 31) or whether they have distinct neural bases. One assumption which is often made in the literature might in part explain the contradictory VOD findings, and that is whether ADHD and ASD are dimensional conditions that grade from the general population into the clinical range – i.e. that clinical and non-clinical groups of participants differ quantitatively but not qualitatively. Despite the categorical model advised by the DSM-5 criteria above (1), the last 10 years have seen an increase in reliance on dimensional models in both medical practice and theoretical settings, positing them as more appropriate to traditional categorical systems, as they better capture individual variability, address overlapping and subthreshold presentations, and reflect the population-level distribution of neurodevelopmental features rather than arbitrary diagnostic boundaries (32). The assumption that ADHD and ASD are dimensional constructs is driven by studies such as Ogundele and Morton (33) who report a subthreshold level condition for ADHD and ASD. Genetic studies also offer evidence that such traits grade into the general population, with clinical cases clustering at the extreme end of this continuum (34, 35).

The concept of dimensionality naively assumes that clinical cases are just more severe versions of non-clinical cases. However, non-clinical groups in experimental studies are usually defined simply according to their scores on the relevant scales measuring ADHD and ASD-like traits, whilst a clinical diagnosis is a considerably more comprehensive evaluation of a person’s symptomology, requiring a detailed synthesis of symptom presentation, clinical interview, developmental history, and functional impairment, often integrating information from standardized tools (e.g., ASRS [Adult ADHD Self Report Scale] for ADHD, ADOS [Autism Diagnostic Observation Schedule], ADI-R [Autism Diagnostic Interview- Revised] for ASD) along with direct observation, informant reports, and clinician judgment (36, 37). As a consequence, clinical diagnosis will be sensitive to aspects of the disorders not covered by standardized measures. For example, emotional dysregulation is a widely recognized symptom of ADHD (e.g. 38), but does not feature in the ASRS. Therefore, whilst the measured traits may or may not be dimensional, there are reasons to consider that clinical and non-clinical groups to be qualitatively different because clinical groups present with greater symptom complexity while non-clinical groups are typically defined purely by their high scores on symptom scales. As a consequence, it is possible that contradictory findings in the literature arise when considering the results of studies using non-clinical samples (as with 26, 29) versus clinical samples (as with 28) because the disorder is ‘diagnosed’ in different ways. The picture is further complicated by the fact that – given the prevalence of undiagnosed cases of adult ADHD and ASD (39, 40) - ‘non-clinical’ groups may actually contain participants who should have had a clinical diagnosis and hence may be qualitatively different to truly non-clinical cases. The extent to which that is true will potentially affect the outcome of the study.

For this reason, in the current study we aim to explore these issues by comparing VOD in clinical and non-clinical ADHD and ASD cohorts who are matched on the core characteristics of their disorder using standardized tools. The assumption is that clinical diagnosis will have independent weight in relation to sensory performance and therefore clinical cohorts across the two conditions will perform differently to non-clinical cohorts in terms of orientation sensitivity, although the direction of the difference cannot be predicted *a priori*. The results will hopefully help to explain why some previous research using visual discrimination performance found that ASD cohorts outperform with increases in severity (28, 29) but other researchers found either no relationship between performance and severity (30) or a decrease in performance with increased severity (26). In addition, we will extend the clinical vs non-clinical comparison to ADHD cohorts as well.

## Methods

### Participants

Descriptive statistics are available in **Table 1**. A total of 158 participants took part in the study, of whom 6 were excluded due to incomplete task or questionnaire data. The final sample consisted of 152 participants, divided into four groups (n = 38 per group): individuals with a clinical diagnosis of ADHD or clinical diagnosis of ASD, and their matched non-clinical counterparts. Of these, 88 identified as female, 59 as male, and five as non-binary. The majority of participants (60%) identified as White European/British/Irish with a college-level education, while the remaining 40% represented diverse ethnic backgrounds, including Asian, Black, and Mixed Ethnicity. Ages ranged from 18 to 57 years. The non-clinical cohort were recruited from the University of Sheffield and the surrounding community. For consistency with prior literature, we use the term ‘*non-clinical’* to refer to participants without a formal clinical diagnosis of ADHD or ASD. This terminology is used operationally to indicate diagnostic status only and does not imply diagnostic negativity.

**Table 1:** Descriptive statistics for the clinical and non-clinical matched cohorts showing the number of participants (N) in each group, the gender (Male and Female) split, mean age, standard deviation (SD) of the ages and the age range.

Group	N	Gender	Mean age	SD age	Age range
ADHD Clinical	38	Female: 23 (60.5%),	32.45	10.74	18–57
		Male: 14 (36.8%)			
ADHD Non-Clinical	38	Female: 24 (63.2%),	25.32	8.24	18–42
		Male: 14 (36.8%)			
ASD Clinical	38	Female: 24 (63.2%),	30.74	11.04	18–57
		Male: 14 (36.8%)			
ASD Non-Clinical	38	Female: 25 (65.8%),	21	5.04	18–42
		Male: 13 (34.2%)			

Participants in the clinical cohort were recruited through National Health Service (NHS)-affiliated support services groups and NHS-connected local councils’ neurodiverse groups for individuals with ADHD and ASD. Participants had an existing clinical diagnosis of ADHD or ASD established prior to participation and evidence of clinical diagnosis was provided at enrolment. Consistent with standard practice in adult ADHD and ASD research, participants were not re-assessed diagnostically for the purposes of the study, and clinical diagnosis was treated as the baseline criterion for inclusion in the clinical cohorts (41). Based on scores on standardized measures used in this study (see below), participants from the ADHD clinical cohort were predominantly of the inattentive phenotype, followed by combined and hyperactive phenotypes, in line with other research within adult populations (42, 43), while the ASD clinical cohort scored highest on difficulties within the social communication domain, followed by repetitive behaviors, in line with Leford-Besnard et al. (44). In the clinical ADHD cohort, 20 participants reported current use of ADHD-related medication. In contrast, none of the ASD clinical cohort or any other participants reported psychotropic medication use. Finally, comorbid conditions such as anxiety, depression and learning disabilities were reported by many participants across the clinical cohorts and are detailed in **Supplementary Table S1**. These were not modelled as independent variables, as contemporary transdiagnostic frameworks (e.g., HiTOP, p-factor models [45, 46]) conceptualize comorbidity as arising from shared latent dimensions rather than from independent, separable disorders. Within this perspective, comorbidities are integral to the clinical phenotype and diagnostic process in adult ADHD and ASD, rather than nuisance variance to be controlled.

All participants provided informed consent and received detailed participant information and debrief forms, in accordance with the Declaration of Helsinki (47). Ethical approval was obtained from the University of Sheffield Ethics Committee. Each participant was assigned a unique, anonymized code to maintain confidentiality. This study was preregistered on the Open Science Framework (OSF) prior to data analysis and a link to it can be found in the Preregistration statement below.

### Questionnaires

To match the groups in terms of their scores using standardized tools, the presence of ADHD and ASD traits were assessed using two self-report measures specifically designed to assess dimensional psychiatric traits in adults, administered via the online platform Qualtrics XM (Qualtrics, U.S.A.) and presented in a randomized order. ADHD traits were assessed using the Adult ADHD Self-Report Scale (ASRS; 37), and ASD traits were measured using the Broad Autism Phenotype Questionnaire (BAPQ; 48).

The ASRS is an 18-item instrument based on the DSM-IV diagnostic criteria for ADHD, comprising two subscales—Inattention (IN) and Hyperactivity/Impulsivity (HP)—each containing nine items. Responses are rated on a five-point Likert scale (Never, Rarely, Sometimes, Often, Very Often) indicating how frequently each statement applied to participants over the past six months (e.g., IN: “How often do you have problems remembering appointments or obligations?”; HP: “How often do you interrupt others when they are busy?”). In the current study Cronbach’s alpha was above.87.

The BAPQ consists of 36 items divided into three subscales—Aloof Personality (AP), Pragmatic Language (PL), and Rigid Behavior (RB). Participants rated how characteristic each statement was of them during the preceding six months using a six-point Likert scale (Very Rarely, Rarely, Occasionally, Somewhat Often, Often, Very Often). Example items include: AP – “I would rather talk to people to get information than to socialize,” PL – “It’s hard for me to avoid getting sidetracked in conversations,” and RB – “I am comfortable with unexpected changes in plans.” In the current study Cronbach’s alpha was above.70.

### Orientation discrimination task

**Figure 1:**
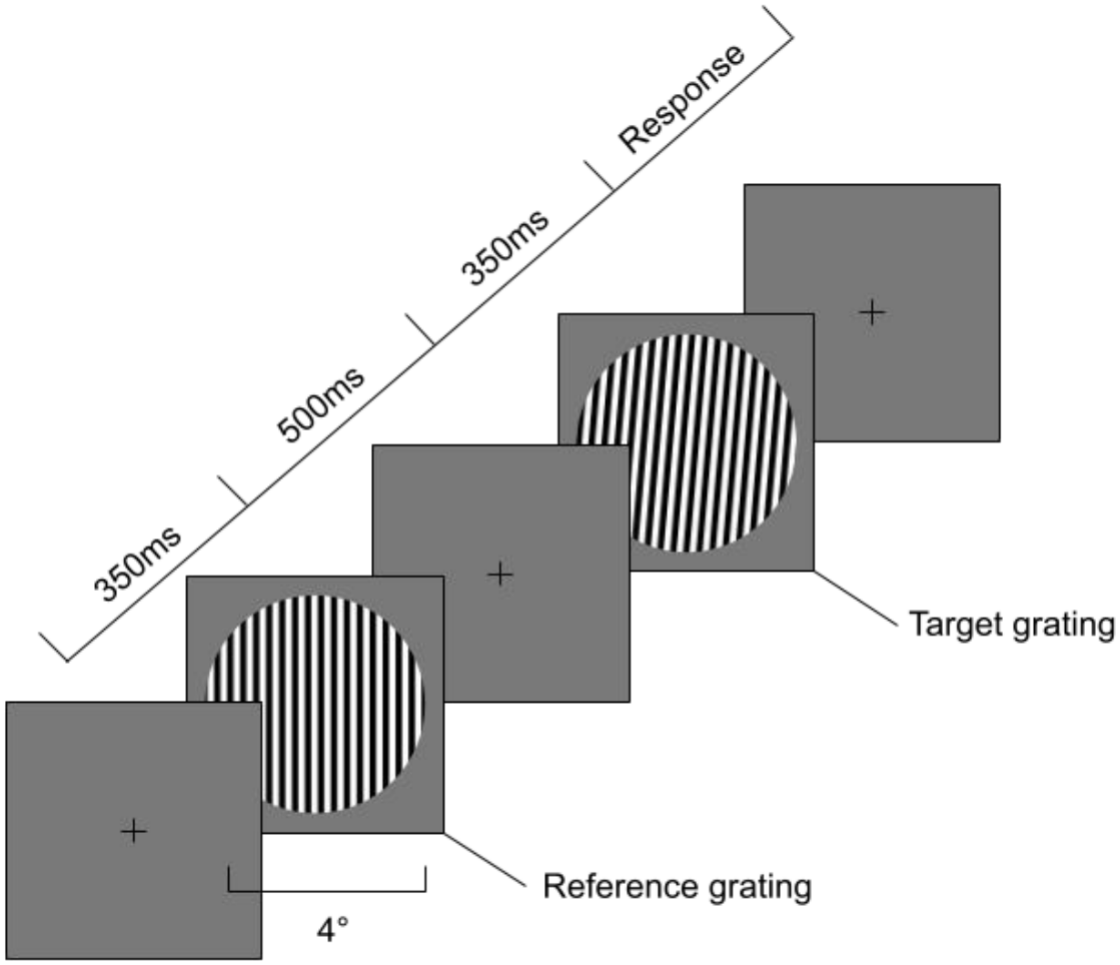
Sequence of events during the visual orientation discrimination task. First, a 350 milliseconds (ms) reference grating is presented at either a vertical or oblique angle with a diameter of 4 degrees of visual angle. Second, after an interstimulus interval (with a fixation cross) of 500ms a target grating is presented a number of degrees away from the reference grating with either a clockwise or anti-clockwise rotation.

Visual orientation discrimination thresholds were measured using a method of constant stimuli combined with a two-alternative forced-choice (2AFC), adaptive, and randomly interleaved staircase procedure, following the one-up–three-down design described by Dickinson et al. (29) and Edden et al. (49). On each trial, participants viewed a reference sinusoidal grating followed by a target grating, each presented for 350 ms and separated by a 500 ms interstimulus interval. Their task was to indicate whether the target grating was rotated clockwise or anticlockwise relative to the reference grating by pressing the right or left arrow key, respectively (see **Figure 1** for trial sequence).

Stimuli were generated using PsychoPy v2022.2.4 and presented in a mixed sequence, such that participants were never exposed exclusively to vertical or oblique orientations. Each grating subtended 4° of visual angle (4 cm diameter), had a spatial frequency of three cycles, mean luminance of 45 cd/m², and 80% contrast. Stimuli were displayed on a linearized LCD monitor viewed through a circular aperture to eliminate orientation cues from the screen edges. A chinrest positioned 57 cm from the monitor stabilized head position. Gamma and luminance calibration were performed using PsychoPy’s gamma correction utility and a photometer.

The target grating initially differed from the reference by 5° and adapted according to performance: the orientation difference decreased after three consecutive correct responses (increasing task difficulty) and increased after one incorrect response (decreasing task difficulty) until nine reversals occurred. After each reversal, the step size was reduced to 75% of its previous value. Orientation differences ranged from 0.001° to 20° relative to the reference. The task comprised two conditions: a vertical condition (reference = 0°) and an oblique condition (reference = 45°). For each condition, two staircases tracked clockwise and anticlockwise rotations, resulting in four interleaved staircases (0°clockwise, 0° anticlockwise, 45°clockwise, 45° anticlockwise). Staircases converged on 79% correct performance (50).

### Procedure

An information sheet was provided, and written consent was obtained from all participants prior to beginning the study. Participants completed the battery of questionnaires up to 24 h before attending the testing session for the visual task. Upon arrival, they were asked to store away any light-emitting devices and remained in the testing room for 30 min to allow for dark adaptation, during which all light sources were switched off and monitor covers were placed in position [following Kalloniatis & Luu, (51)]. The visual task comprised two main sections: a practice session, followed by a 40 s rest period, and the main task, which was divided into blocks with 2 min breaks between them and lasted approximately 17 min in total. After completing the task, participants were debriefed and given the opportunity to ask questions about the study.

### Data analysis

#### Power analysis

*A priori* power analysis was conducted using G*Power 3.1 (52) to determine the required sample size for the planned ANCOVA models comparing two diagnostic groups (clinical vs non-clinical) with two covariates (age and gender). Assuming a medium effect size (f = 0.25, corresponding to partial η² ≈.06), an alpha level of.05, and desired power of.80, the analysis indicated that a minimum of 128 participants (64 per diagnostic comparison; 32 per group) would be required to detect significant group differences after controlling for the covariates.

#### Propensity matching and validation

To minimize potential confounds arising from naturally occurring group differences, we used propensity score matching (PSM) to create statistically comparable clinical and non-clinical cohorts prior to analysis, selecting matched non-clinical participants from a larger pool of community respondents based on their ASRS scores (for ADHD) and BAPQ scores (for ASD) (53). Because group membership (clinical vs. non-clinical) was known in advance, logistic regression was not used to classify participants but to estimate each individual’s propensity score—the predicted probability of belonging to the clinical group. Separate models were estimated for the ADHD and ASD samples using the logit of the predicted probability as the propensity score. Matching was performed via Python using a 1:1 nearest-neighbor algorithm without replacement, constrained by a caliper width of 0.20 standard deviations of the logit-transformed propensity score to ensure high-quality matches within the region of common support. When multiple potential matches met the caliper criterion, the pair with the smallest absolute logit distance was selected. Matching was conducted independently for the ADHD and ASD datasets; therefore, limited overlap in non-clinical controls across the two matched sets was permitted when criteria were satisfied. Balance was evaluated using standardized mean differences (target |SMD| < 0.10), independent-samples t-tests, and visual inspection of propensity score distributions. This procedure yielded four well-balanced groups (n = 38 per group; see **Table 1**): Clinical ADHD, non-clinical ADHD (matched controls), clinical ASD, and non-clinical ASD (matched controls). Post-matching validation confirmed no significant group differences in trait measures: for ADHD, a Mann–Whitney U test indicated no difference in ASRS scores, *U*(74) = 699.5, *p* = .819, *r* = .03; for ASD, an independent-samples t-test revealed no difference in BAPQ scores, *t*(74) = –0.37, *p* = .713, *d* = –0.09. All subsequent inferential analyses (ANOVAs and ANCOVAs) were conducted on the matched samples. The full Python code implementing the PSM workflow is provided in the **Supplementary Materials**, a link to which can be found in the Data Availability Statement.

#### Data analysis

All analyses were conducted in JASP. Data were first screened for completeness and accuracy: participants with incomplete task or questionnaire data were excluded, and all variables were inspected for range violations, outliers, and missingness. For the psychophysical thresholds, mean discrimination values were computed separately for the vertical and oblique conditions for each participant. Distributions were visually inspected using Q–Q plots, and normality was statistically evaluated with Shapiro–Wilk tests. Across all key variables (thresholds and trait measures), the tests indicated that data were approximately normally distributed (*p* >.05). Homogeneity of variances, assessed using Levene’s tests, was also satisfied in all main analyses (*p* >.05), confirming that assumptions for parametric testing were met. Outliers were screened using ±3 SD and evaluated for undue influence; no extreme values required removal. All analyses were conducted using a two-tailed α = .05, and effect sizes were reported as η² or partial η² with 95% confidence intervals.

Although the clinical and non-clinical groups were matched on the critical variable of their ASRS or BAPQ scores, given the between group differences in age and gender (see **Table 1**) our primary models were ANCOVAs (analysis of covariance), controlling for age and gender as covariates. ANCOVA assumptions were checked in JASP: linearity between covariates and the outcome, homogeneity of regression slopes (Group × Covariate interactions), normality of residuals, and homoscedasticity. Where assumptions were infringed, we report the appropriate robust alternative or note the limitation. For each ANCOVA, we report the F statistic, p value, partial η², and adjusted means with 95% CIs.

## Results

### ANCOVA results- ADHD

#### Vertical thresholds

All ANCOVA results are presented in **Table 2**. The averaged vertical threshold of the ADHD clinical group was 1.51 (degrees of rotation), while for the non-clinical group it is 1.86. An ANCOVA controlling for age and gender revealed no significant difference in vertical orientation thresholds between the ADHD clinical and non-clinical groups, *F*(1,72) = 0.59, *p* = .444, partial η² = .008. The non-clinical group exhibited slightly higher thresholds (*M* = 1.90, *SD* = 1.30) than the ADHD group (*M* = 1.52, *SD* = 1.10), indicating marginally poorer performance, but this difference was not statistically meaningful. Both covariates were non-significant (*p*s >.15).

**Table 2:** ANCOVA results for the diagnostic groups (ASD, ADHD) and measures (vertical and oblique thresholds).

Diagnostic group	Measure	F(1,72)	p	Partial η²	Clinical M (SD)	Non-clinical M (SD)	Direction of effect
ADHD	Vertical threshold	0.59	0.444	0.008	1.52 (1.10)	1.90 (1.30)	n.s. (Non-clinical > Clinical)
	Oblique threshold	4.11	0.046	0.054	5.38 (2.90)	4.05 (2.53)	Clinical > Non-clinical
ASD	Vertical threshold (Welch-corrected)	4.33¹	.041 (.016¹)	0.057	1.37 (0.73)	2.00 (1.40)	Clinical < Non-clinical
	Oblique threshold	1.58	0.212	0.022	4.45 (2.81)	3.90 (2.48)	n.s. (Clinical > Non-clinical)

The table shows the clinical and non-clinical means (M) and standard deviations (SD), and the direction of effect (with relevant statistics: F-test (1,72), significance (p), partial η²- effect size).

#### Oblique thresholds

The averaged oblique threshold for the ADHD clinical group was 5.38, while for the non-clinical group it was 4.05. A separate ANCOVA indicated a significant main effect of diagnostic group, *F*(1,72) = 4.11, *p* = .046, partial η² = .054, with individuals in the ADHD clinical group (*M* = 5.38, *SD* = 2.90) showing reduced sensitivity to oblique orientations compared with matched controls (*M* = 4.05, *SD* = 2.53). Neither age (*p* = .875) nor gender (*p* = .950) significantly predicted performance.

Although 20 of the 38 participants in the ADHD clinical group were receiving medication for their condition, medicated (vertical: *M* = 1.50; oblique: *M* = 5.44) and unmedicated participants (vertical: *M* = 1.53; oblique: *M* = 5.37) did not differ significantly for either vertical, *t*(35.80) = −0.49, *p* = .626, or oblique thresholds, *t*(35.91) = 1.14, *p* = .262.

### ANCOVA results- ASD

#### Vertical thresholds

The averaged vertical threshold for the ASD clinical group was 1.31, while for the non-clinical it was 2.00. An ANCOVA controlling for age and gender revealed a significant main effect of group, *F*(1,72) = 4.33, *p* = .041, partial η² = .057. Participants with clinical ASD (*M* = 1.37, *SD* = 0.73) outperformed non-clinical controls (*M* = 2.00, *SD* = 1.40), indicating enhanced vertical orientation sensitivity. The assumption of homogeneity of variance was violated (Levene’s *p* = .010); therefore, results were verified with a Welch correction, which produced an equivalent outcome (*F* = 6.14, *p* = .016). Covariates were non-significant (*p*s >.33).

#### Oblique thresholds

The averaged oblique threshold for the ASD clinical group was 4.45, while for the non-clinical group it was 3.90. There was no significant difference in oblique thresholds between groups, *F*(1,72) = 1.58, *p* = .212, partial η² = .022. The ASD clinical group (*M* = 4.45, *SD* = 2.81) performed comparably to non-clinical controls (*M* = 3.90, *SD* = 2.48), with residuals meeting normality assumptions and Levene’s test indicating equal variances (*p* = .457). Both covariates were non-significant: age (*F*(1, 72) = 1.17, *p* = .283, partial η² = .016) and gender (*F*(1, 72) = 0.41, *p* = .525, partial η² = .006), indicating that neither variable significantly predicted oblique threshold performance.

## Discussion

The present study is the first to not just compare ADHD and ASD but to also compare the performance of clinical versus non-clinical cases in an effort to clarify whether *visual sensory performance*, as indexed by orientation discrimination, differs between ADHD and ASD in a manner that may reflect disorder-specific processing profiles. Specifically, we compared VOD performance in clinical and non-clinical cohorts matched on standardized self-report measures to test the impact of diagnosis on visual orientation discrimination. Previous research has shown inconsistent findings in relation to the above. Some have reported enhanced visual discrimination performance in ASD (28, 29), whilst others have found no significant relationship between non-clinical ASD traits and visual discrimination thresholds (30). Our own earlier work (26) for a first time investigated both ADHD and ASD within the same cohort and suggested, in contradiction to Bertone et al. (28) and Dickinson et al. (29), comparable sensory impairments between the two conditions but with distinct, disorder specific, trajectories.

In the current study, across both diagnostic groups, orientation discrimination performance showed distinct patterns. In the ADHD sample, individuals with a clinical diagnosis presented with poorer performance than the matched non-clinical cohort as indicated by their higher scores and hence reduced sensitivity to oblique orientations compared with matched non-clinical controls, while vertical thresholds did not differ significantly between groups. In contrast, the ASD clinical group demonstrated better vertical orientation sensitivity than their non-clinical counterparts as indicated by their lower scores on the vertical task. These effects remained after controlling for age and gender in both samples, and all assumptions of normality and homogeneity were met. Overall, the results indicate that ADHD and ASD are each associated with subtle but distinct alterations in orientation discrimination—ADHD linked to weaker (oblique) sensory performance and ASD to enhanced (vertical) sensory discrimination. Our results also suggest that medication did not have an impact on the performance of the ADHD clinical participants, suggesting low-level processing might not be affected by ADHD-related stimulants.

### The clinical group differences indicate two divergent profiles

The differential performance of clinical and non-clinical ASD participants suggests that those individuals with a clinical diagnosis may be qualitatively different to those without, in spite of their match in terms of their standardized clinical characteristics. As we mentioned in the introduction, a clinical diagnosis is a considerably more comprehensive evaluation of a person’s symptomology, requiring a detailed synthesis of symptom presentation, clinical interview, developmental history, and functional impairment, often integrating information from standardized tools along with direct observation, informant reports, and clinician judgment (36, 37). Such a comprehensive evaluation will potentially detect disorder specific characteristics that are not encapsulated in commonly used report tests like the BAPQ. The fact that clinical and non-clinical ADHD participants differed only in terms of the severity of the deficit, in contrast to the clinical ASD participants who outperformed the non-clinical ASD participants, suggests that the two conditions may differ in how clinical diagnosis modulates *visual perceptual processing*, even when trait severity is held constant.

These disorder-specific trajectories are consistent with two previous studies which researched the extent to which sensory processing is comparable in the two conditions (26, 54). In the first study, the two conditions formed distinct interpersonal and intrapersonal clusters of behaviors in a network analysis: ADHD traits were linked to inattention and difficulties maintaining a train of thought, whereas ASD traits were uniquely associated with social anxiety—often underpinned by an increased focus on detail over context (55). Similarly, in the second study, non-clinical cohorts with ADHD and ASD traits were tested on a VOD task. Although both groups showed similarly impaired performance (elevated thresholds), visual performance was linked to anxiety in the ADHD cohort but not the ASD cohort, again distinguishing the two conditions. These distinct profiles are supported by evidence that ADHD-related impairments arise from alterations in sustained attention and working memory (56, 57), whereas impairments in ASD appear to reflect a cognitive style characterized by heightened sensitivity to fine-grained features and subtle differences in stimuli, but reduced ability to integrate these into a global whole (58–60).

An important question is the extent to which our findings have implications for the idea that ADHD and ASD are dimensional disorders. At a descriptive level, our results are broadly consistent with the notion that ADHD aligns more closely with a dimensional account, insofar as the clinical group showed greater impairment than the non-clinical group while differing primarily in degree rather than in kind. This pattern is compatible with models in which ADHD traits are continuously distributed, with measures reflecting a broad factor structure and clinical and non-clinical individuals occupying different positions along that continuum (see 61–63). At the same time, the nature of this dimensionality remains ambiguous. This is particularly relevant given that the ASRS does not capture several clinically salient features of ADHD that may contribute to group differences. In contrast, the pattern observed for ASD appears less readily accommodated by a simple dimensional account. Clinically diagnosed participants demonstrated superior performance relative to high-trait, non-clinical individuals, and this finding complements prior evidence of a negative association between autistic traits and visual discrimination thresholds in non-clinical samples (26). Taken together, these observations suggest that increasing autistic trait levels do not map monotonically onto sensory performance in the same way across clinical and non-clinical groups. Rather than implying a strictly categorical distinction, this pattern is consistent with models proposing a hybrid structure for ASD, in which continuously distributed traits coexist with qualitative clustering of core features—particularly within social-communication, repetitive behavior, and sensory domains—that distinguish clinically diagnosed individuals from high-trait non-clinical cases (64). From this perspective, a clinical ASD diagnosis may reflect a distinct configuration and organization of traits and functional impacts, rather than simply a higher position on a single continuum of autistic characteristics. Consequently, approaches that integrate both dimensional and categorical perspectives may be better suited to capturing the heterogeneity and boundary structure of ADHD and ASD, as well as the risk of overlooking atypical or subthreshold presentations when relying on either framework alone (65, 66).Further research across all sensory modalities is needed to ascertain nosological patterns.

### What do these results mean for the impact of clinical diagnosis

The finding that clinical groups in both conditions performed differently to non-clinical groups bring us back to the question of the impact of a clinical diagnosis, which appears to depend on more than just passing a certain threshold score of severity. Our results within the context of visual sensory performance and more specifically the ability to visually discriminate in ADHD and ASD, suggest that there seems to be a qualitative component to diagnosis which captures not just severity of traits but the impact of core aspects of the disorder on daily life which are unique for the clinical participants. In recent years issues with self-reported and self-diagnosed ADHD and ASD have been raised by clinical researchers (such as 67–69) in line with the notion that it is possible unique trajectories and patterns of behavior are observed in both self-diagnosed and clinician diagnosed cohorts. Banker et al. (67, 70) report a significant lack of agreement between the self-rated and clinician-assessed symptoms in groups of clinical and self-rated ASD cohorts, suggesting symptoms reported in each of the two groups should be interpreted separately rather than as gradient of magnitude. Further research is needed to understand whether and how clinical diagnosis could differentiate ADHD and ASD across cognitive or sensory domains.

The unique formulation of impact coming from a clinical diagnosis highlights that diagnosis goes beyond what is revealed by simply increasing or decreasing trait severity scores. Clinicians, as discussed above, use multidimensional assessment focusing on functional impact and nuanced symptoms, which allows for the identification of distinctive features in clinical cases. Whilst the BAPQ may capture traits like aloofness, rigidity, and pragmatic language issues, it does not adequately detect the subtle qualitative differences in social reciprocity, nonverbal communication or adaptive/masking behaviors that can characterize ASD (71), particularly in adults and which play a crucial role in the diagnostic process (67). Clinical interviews and comprehensive assessments integrate these missing dimensions via direct observation, collateral information, real-world history, and probing for contextual factors. This richer, qualitative, multi-informant evaluation allows clinicians to identify the presence, configuration, and impact of symptoms beyond what questionnaires alone can capture, ensuring a more accurate and ecologically valid diagnosis. Such assessment in relation to ASD might allow diagnostic processes to systematically differentiate people whose local bias and enhanced sensory discrimination are core, enduring aspects of their functioning from those who merely score high on questionnaires but lack the persistent, generalizable pattern or real-world impact needed for a clinical diagnosis.

Likewise, in relation to ADHD, the ASRS primarily assesses the core symptoms - inattention, hyperactivity, and impulsivity, however, it misses key aspects crucial to the configuration of the condition, such as emotional dysregulation (e.g., irritability, mood swings, frustration intolerance), difficulties with time management and organization, and variability across contexts, all of which are often clinically significant and may contribute as much, if not more, to daily impairment as the core ADHD features but are yet under-recognized by the scale alone (72–74).

### Conflicting previous findings for sensory processing and ASD

While previous findings concerning low level perception in ADHD are scarce, research on ASD and sensory processing has produced conflicting results in the context of VOD. As discussed above, some previous research using visual discrimination performance found that ASD cohorts perform better with increases in severity (28, 29) but other researchers found either no relationship between performance and severity (30) or a decrease in performance with increased severity (26). Although contradictory, the results of the current study may offer insight into the basis of the contradiction. We have found that individuals with a clinical diagnosis have enhanced VOD performance, which is also supported by the findings of Bertone et al. (28). The contradictory finding come from studies that have used participants they’ve classed as ‘non-clinical’. One possibility, again suggested by our findings, is that for a cohort that is truly non-clinical (i.e. would not be diagnosed as ASD by a clinician), VOD performance is impaired as in our earlier work (26). However, other studies using ostensibly non-clinical cohorts may have inadvertently recruited individuals who would have attracted a clinical diagnosis under the correct circumstances. The extent to which the tested sample contained such individuals could turn impaired performance into enhanced performance (as in 29) or simply offset impaired performance to arrive at overall neutrality (as in 30).

In view of the points discussed above, the major contribution of our findings is that they demonstrate - in spite of high level similarities in sensory processing abnormalities in ASD and ADHD - the conditions differ at a more granular level of analysis. Although ASD and ADHD can be conceptualized as related in their sensory impairments, they do not seem to be identical. Within the visual sensory domain examined here, ADHD and ASD showed different patterns of diagnostic modulation on this orientation discrimination measure that are *consistent with* dimensional and hybrid interpretations, respectively, though investigation over various aspects of sensory processing and across all modalities is needed to understand more definitively disorder specific structures. We cannot therefore postulate that they arise from a common neural substrate, at least in terms of sensory processing component to the disorders. We also demonstrate that the impact of a clinical diagnosis is far more nuanced than simply representing a point of higher severity on a continuous trait scale; rather, clinical diagnosis identifies qualitative and core differences in symptom profiles, functional impact, and neurocognitive organization that are not captured by rating scores alone.

### Limitations

Although we assess sensory processing within the visual modality in this study, we did not consider sensory differences in other modalities, which may diverge in patterns and relevance. This is important in order to ascertain the full picture of sensory functioning. In addition, as some research has suggested different profiles of impairment in ADHD between adolescent and adult cohorts (75), it is important to look at younger ages and ascertain whether the same clinical impact on behavior will hold. Further, this study does not discuss the impact of comorbidities. The important impact comorbidities have on sensory processing has already been described in non-clinical cohorts of ADHD and ASD (26) and an assessment of their impact in clinical populations is needed in adults, however this is beyond the scope of the current paper. Finally, conclusions regarding dimensional versus categorical models are necessarily limited by the use of a single perceptual task within one sensory modality and should be interpreted within the context of the current research. Larger cohorts and examination across all modalities is to needed to evaluate these dimensional–hybrid interpretations more robustly.

## Preregistration statement

The study pre-registration page on the OSF website plan can be found at 10.17605/OSF.IO/N7X3B.

## Data Availability

Researcher materials and analysis code are available at 10.15131/shef.data.30704810.
