# Supplementary material for: Visual orientation discrimination in adults with ADHD and ASD: the differential impact of clinical diagnosis and trait severity
Source: Front Psychiatry. 2026 Feb 23;17:1754032. doi: 10.3389/fpsyt.2026.1754032 (PMC12968783; doi:10.3389/fpsyt.2026.1754032)
Supplement: Supplementary file 1 [file Table1.docx]

| **Comorbid condition** | **ADHD (n)** | **ASD (n)** |
| --- | --- | --- |
| Social anxiety disorder | 19 | 23 |
| Generalized anxiety disorder | 21 | 23 |
| Panic disorder | 17 | 18 |
| Depression | 17 | 19 |
| Bipolar disorder | 1 | 1 |
| Learning disabilities | 8 | 8 |

Supplementary Table S1. Self-reported psychiatric comorbidities by diagnostic group
